# Cardiovascular Disease-Related Lifestyle Factors and Longevity

**DOI:** 10.4061/2011/386892

**Published:** 2011-12-29

**Authors:** Christina Chrysohoou, Christodoulos Stefanadis, Christos Pitsavos, Demosthenes Panagiotakos, Undurti N. Das, Dario Giugliano

**Affiliations:** ^1^First Cardiology Clinic, School of Medicine, National and Kapodistrian, University of Athens, 11528 Athens, Greece; ^2^Department of Nutrition and Dietetics, Harokopio University, 17671, Athens, Greece; ^3^Jawaharlal Nehru Technological University, Hyderabad, Kakinada 533003, India; ^4^Second University of Naples, 80138 Naples, Italy

Cardiovascular disease is one of the leading causes of death in the developed world. Understanding trends in cardiovascular disease prevalence and identifying factors associated with its development are important, since they provide information to define the burden and the mechanisms of the disease, which is the first step before population-based interventions. This special issue is devoted to highlight lifestyle factors and behaviors associated with the development of cardiovascular disease. According to the World Health Organization, sedentary lifestyle, smoking habits, and unhealthy dietary habits, are the most important factors associated with the development of cardiac diseases; and the main reason for classifying these factors as the most significant is because they can be prevented. 

In their paper, G. S. Metsios et al. presented current scientific evidence about the role of passive smoking in the development of cardiovascular disease in children. The authors identified a total of 42 articles (i.e., 30 reviews and 12 observational), and they revealed that passive smoking may be implicated in deteriorating cardiovascular status in children in terms of unfavorable high-density lipoprotein levels and deteriorated vascular function. In another article based on a middle-aged population of the Ikaria island in Greece, C. Chrysohoou et al. reported that short-term depressive symptoms are related to a worse 30-day prognosis of ACS patients; however, this relationship was mediated by Mediterranean diet adherence. It is well known that depression is an independent risk factor for cardiac diseases. Due to a significant increase of the incidence of depression during the past decades, there has been a growing interest regarding the mechanisms underlying its pathogenesis, including the parameter of nutrition. Y. Ma et al. reported that chronic depression is associated with inflammatory response in women, but not in men. Previous studies have also observed the positive relationship between depression and inflammation. It seems that Mediterranean diet, due to its antioxidant role, may mediate the adverse effect of depression on cardiac system; the majority of studies suggest the beneficial effects of omega-3 PUFAs, a main constituent of Mediterranean diet, in the treatment of clinical depression, with eicosapentaenoic acid (EPA) being more effective than docosahexaenoic (DHA). Another lifestyle factor that has received much attention concerning the prevention of cardiovascular diseases is physical activity status. In the paper by S. A. Kavouras et al., from the ATTICA study, the investigators revealed another mechanism about the protective role of physical activity in atherosclerosis, the improvement in total antioxidant capacity of the study's participants. Moreover, under the context of the IKARIA study, the authors observed that frequent fish intake (i.e., >3 times per week) seems to moderate depressive mood. In addition to the latter, Tyrovolas et al. reported that nutrition services provided in Greek islands played a significant role in modifying dietary habits of older adults. M. M. Pryde and W. B. Kannel resumed that dietary modifications about polyunsaturated fat intake, processed meat consumption, fish choices and preparation, trans fatty acids, low carbohydrate diets, egg consumption, coffee, added sugar, soft drink beverages, glycemic load, chocolate, orange juice, nut consumption, vitamin D supplements, food portion size, and alcohol drinking may preserve cardiovascular health and longevity. 

In this special issue, the role of lifestyle, mainly represented by diet, smoking, and physical activity, has been extensively studied in relation to cardiovascular disease development. The scientific evidence strongly supports the protective role of healthy diet, abstinence of smoking, and engagement in physical activities for the prevention of cardiovascular disease. Public health policy makers should target on these lifestyle behaviors which seemingly can cost-effectively reduce the burden of the disease at population level. 

*Christina Chrysohoou* *Christodoulos Stefanadis* *Christos Pitsavos* *Demosthenes Panagiotakos* *Undurti N. Das* *Dario Giugliano*

